# Dr. Williams on Auscultation

**Published:** 1829-04-01

**Authors:** 


A RATIONAL EXPOSITION OF THE PHYSICAL SIGNS OF THE DISEASES OF THE LUNGS AND PLEURA; ILLUSTRATING THEIR PATHOLOGY, AND FACILITATING THEIR DIAGNOSIS. By *Charles J. B. Williams*, M.D. 8vo. pp. 192. 1828.

SINCE the great work of Laennec was published, several attempts have been made to abridge the study of auscultation, by means of manuals; but none of them have answered the purpose. They were too brief. Dr. Williams has come to the task with much acoustic science, and has endeavoured to explain the causes of phenomena, with more precision and on more fixed principles than his predecessors. As we mean to dedicate a long article to affections of the pleura in the next number of this Journal, we shall then have an opportunity of reverting to Dr. Williams' work. At present, we shall do little more than announce and recommend it, offering one or two specimens of the execution.

"The 'Traité de l'Auscultation Mediate,' and the perfect translation of Dr. Forbes, are at length generally appreciated, even in this country, slow to award its meed of praise. The homage paid to the talents of the author, gives me a gratification that almost seems personal; and I doubt not that this feeling is shared by others of his pupils, in whom his urbane and amiable deportment created a sincere regard for the man, as his great mental abilities excited our respect. His great talents are known to the public through the medium of his writings; but those who attended his clinique can alone appreciate the wonderful acuteness of perception, and faculty for observation, that enabled him to carry his discovery to the degree of perfection in which he left it; and they, above all, witnessed, felt, and profited by the solicitous interest which he showed to make others partake of its inestimable advantages. They felt in his death the loss of a friend; as science had to deplore the loss of his talents: he has wrought a good work for both; the feeling shall last while they last; science has recorded his name on her tablets for ever:—

'Illum aget pennâ metuente solvi

'Fama superstes.'

"Let me say a few words on the objects and plan of the present work.

"I have ever found in practice, and it is perfectly conformable to reason, that the easiest and most agreeable way to study physical signs, and to attain the surest criterion of their value and importance, is by considering how they are caused, or what are the relations in which they stand to the physiological and pathological states that produce them. Attempts to discover the rationale of the general symptoms of disease have been as unsuccessful as our knowledge of the functions or properties, on which they depend, is scanty and imperfect; and inquiries of this kind have been proportionately unsatisfactory and unprofitable. But physical signs stand on the broad and intelligible basis of physical laws, and are as readily explained as other simple phenomena, illustrated by natural philosophy. It has been my endeavour to exhibit them, as far as possible, in this intelligible view; to show the mechanism by which the signs are produced, and the manner in which, according to fixed laws, they result as phenomena; to make a knowledge of the pathology predicate the signs, and a knowledge of the signs indicate the pathology; and by thus familiarizing the mind with their principles, to enable it to understand the multifarious forms which, by combination, these signs may assume, and to judge of the corresponding physical changes that modify or produce them." xi.

Dr. Williams had the good fortune of studying in the wards of La Charité, where Laennec taught and Andral prosecuted his labours.

The work is divided into two parts—the first containing an exposition of the general physical signs of a healthy and diseased state and action of the thoracic viscera, to which is prefixed a chapter on the properties, &c. of sound—the second, comprehending the pathological history, and physical signs of the principal diseases of the lungs and pleura. At the end of the work are inserted some *tabular views* of the physical signs, &c. illustrated by a plate, shewing the situation of the regions of the chest. In addition to the above, Dr. Williams has given us a diagram of the stethoscope, and directions for the best principles of its construction. We shall only be able to introduce an extract or two as specimens of the work, in the present article, on account of our confined limits, and because we shall have an early opportunity of again adverting to the publication. Our readers are aware that Laennec considered metallic tinkling as a pathognomonic sign of pneumo-thorax with liquid effusion and fistulous communication with the bronchi. The following fact shews that the presence of a fluid is not essential to the production of this physical sign.

"The following case, which fell under my observation in the ward of M. Lerminier, at La Charité, shews that the presence of liquid effusion is likewise dispensible. A boy, fifteen years of age, had been for some weeks affected with pectoral disease, with cough, shortness of breathing, scanty expectoration, &c., but these had somewhat abated, until a few days before, when they had become considerably aggravated. When I first saw him, he had besides, much fever, quick pulse, pain of side and other symptoms of an acute attack. On percussion the left side sounded well every where, but in the inferior or lateral and posterior region, where it was rather duller than usual. The right side was very sonorous on percussion anteriorly and laterally, below the fourth rib; less so above, and posteriorly. On auscultation, the respiration of the left side was distinct superiorly and anteriorly, but mixed with crepitant rhonchus laterally and posteriorly, particularly in the inferior parts. No bronchophony or bronchial respiration. On the right side the respiration was puerile below the clavicles and in the axilla, and above the spine of the scapula; became less distinct somewhat below, and was quite inaudible below the fourth rib, the part most sonorous on percussion. In this also, after cough and utterance, a distinct metallic tinkling was heard, which appeared not to be affected by change of posture. The next day the same symptoms were present, but the crepitant rhonchus had extended upwards in the left lung; and the patient seemed worse. Tinnitus as before. Died in the course of the day. On dissection, about eighteen hours after death, about the inferior and anterior half of the right side of the chest was found filled with air, some of which escaped with a hissing noise on first incision; the lung was bound down to the whole of the posterior, and the upper portions of the lateral and anterior parietes of the thorax, by a pretty firm fibro-cartilaginous membrane, which also thickened the costal pleura of the cavity. *There was not a drop of liquid in the cavity, and there was no communication with the bronchi.* In the inferior lobes of the compressed lung were found three hydatids contained in, but not connected with, a cavity lined with a fibrous membrane. The tissue of the lung was flaccid, and compressed in their vicinity, and bounding the cavity containing air, but above it was healthy and crepitant. The lower lobes of the left lung were found in a hepatized state, which passed superiorly into simple inflammatory engorgement; and still higher up the tissue was healthy. There were no tubercles in either lung. The rationale of the symptoms is in this case obvious. The tinkling echo was produced in the cavity by the voice, transmitted to it by the pulmonary tissue condensed by the hydatids, and perhaps also by a former liquid effusion in the pleura. This effusion had been absorbed, and the lung being bound down by a factitious membrane, a *pneumothorax of necessity* was left. Such a case of tinnitus metallicus must be very rare, as neither Laennec nor Andral have met with it: and I believe that, although not in all, yet in by far the majority of cases, the organic causes of metallic tinkling are such as Laennec has defined them. The liquid effusion I cannot consider as one of the causes combining to produce it, but merely a necessary accompaniment of its cause, fistulous communication between the pleura and bronchi.

"I have been longer on this article than its importance may seem to require, but the obscurity of the subject demanded it; and if I have shown that the stethoscopic signs are less certian than they have been represented to be, this may prevent future error, and improve our knowledge by guiding us to others more definitive." 146.

### Physical Signs of Hooping-Cough.

"The physical signs of *pertussis* do not materially differ from those of common catarrh, and are usually slight. In the intervals of cough, the respiratory murmur becomes indistinct in some points, and puerile in others; a sibilant of sonorous rhonchus is sometimes heard, and the sound of the chest, on percussion, is unimpaired. From this it may be concluded, that the violence of the cough does not depend entirely on the state of the mucous membrane of the air-passages, and an examination, during a fit of coughing, confirms this conclusion. If the ear is applied to the chest at this period, no rhonchus or respiratory sound is heard, except for a moment, between each cough; and during the sonorous back-draught all is silent within the chest. This absence of the respiratory sound, in an inspiration that seems so deep and forcible, is to be attributed to the admission of air being slow and scanty, on account of the spasmodic constriction of the glottis, by which, too, the hooping noise is caused. A spasm of the muscular fibres of the whole bronchial tract may also contribute to the exclusion of air from the air-cells, but I cannot, with Laennec, consider this as the only cause." 75.

We strongly recommend the work to the attention of auscultators. We must say, however, that it is more adapted to proficients than to learners.

